# Prevalence of Depression, Anxiety, and Stress in Junior High School Students in Guadalajara, Mexico: A Cross-Sectional Survey Study

**DOI:** 10.3390/ijerph192315463

**Published:** 2022-11-22

**Authors:** Manuel Maciel-Saldierna, Emmanuel Elizondo-Hernández, Gabino Cervantes-Guevara, Enrique Cervantes-Pérez, Guillermo Alonso Cervantes-Cardona, Bertha Georgina Guzmán-Ramírez, Irma Valeria Brancaccio-Pérez, Jonathan Matías Chejfec-Ciociano, Mario Jesús Guzmán-Ruvalcaba, Luis Rodrigo Cifuentes-Andrade, Andrea Estefanía Cueto-Valadez, Tania Abigail Cueto-Valadez, Silvia Alejandra Ibarra-Camargo, Mel Paul Mellado-Tellez, Francisco José Barbosa-Camacho, Clotilde Fuentes-Orozco, Alejandro González-Ojeda

**Affiliations:** 1 Secundaria 56 Mixta “Juana de Asbaje”, Guadalajara 44200, Jalisco, Mexico; 2 Departamento de Bienestar y Desarrollo Sustentable, Centro Universitario del Norte, Universidad de Guadalajara, Colotlán 46200, Jalisco, Mexico; 3 Servicio de Medicina Interna, Hospital Civil de Guadalajara “Fray Antonio Alcalde”, Guadalajara 44280, Jalisco, Mexico; 4 Departamento de Disciplinas Filosófico, Metodológicas e Instrumentales, Centro Universitario de Ciencias de la Salud, Universidad de Guadalajara, Guadalajara 44340, Jalisco, México; 5 Departamento de Pediatría, Hospital Civil de Guadalajara Fray Antonio Alcalde, Universidad de Guadalajara, Guadalajara 44280, Jalisco, Mexico; 6 Unidad de Investigación Médica 02, Centro Médico Nacional de Occidente, Instituto Mexicano del Seguro Social, Guadalajara 44349, Jalisco, Mexico

**Keywords:** depression, anxiety, stress, psychological assessment, adolescent

## Abstract

Confinement and a lack of social interaction are associated with depressive symptoms, low self-esteem, and suicidal thoughts. We report the results of a cross-sectional survey of 1414 junior high school students. The aim was to evaluate the prevalence of depression, anxiety, and stress in Guadalajara, Mexico, during the COVID-19 pandemic. Mean scores on the validated Spanish version of the Depression, Anxiety, and Stress Scale (DASS-21) were found to be 6.15 ± 5.6 for depression, 5.8 ± 5.2 for anxiety, and 8.08 ± 5.3 for stress. Female students scored higher in all three conditions (*p* < 0.001). Students who had relatives infected with COVID-19 showed significantly more anxiety than those who did not (*p* < 0.004). Although certain demographic groups are at higher risk of manifesting depression, anxiety, and stress, the student population has also been affected by the global impact of the pandemic.

## 1. Introduction

In recent years, there has been an increase in the prevalence of adolescent psychological disorders, particularly depression, anxiety, and stress. Despite their clinical importance, these emotional disorders have not been adequately addressed. In the United States, Brazil, and Europe, it has been reported that more than 15% of the total population has suffered from depression. Meanwhile, in Mexico, 9.2% of the population has had affective disorders, and 4.8% has experienced a disorder in the previous 12 months. According to the National Survey of Psychiatric Epidemiology, Colombia and Chile have demonstrated similar prevalence [1,2].

Globally, depression and anxiety are the most common disorders. It is estimated that 10% of the population will suffer from a depressive disorder [3] and 33% will suffer from anxiety at some time in their lives [4], with a significant prevalence between the ages of 18 and 25 years [5].

It is clear that the current coronavirus (COVID-19) pandemic has had a global effect with high rates of transmission, death, and adverse impacts on mental health. The world’s population is enduring highly stressful conditions that increase the risk of developing anxiety and depression during outbreaks of COVID-19 [6]. One study evaluated the mental health burden of the COVID-19 pandemic on the general population in China and found that anxiety disorders and depressive symptoms affected 35% and 20% of the population, respectively [7]. A further Chinese study [8] found that 35% of respondents were in psychological distress. Other studies conducted during the COVID-19 period showed a prevalence of 33% and 18% for depression and anxiety, respectively, in Italy [9] and 23% and 45% in Turkey [6].

Isolation, which includes lack of social contact and confinement in a closed space, is associated with increased depressive symptoms, low self-esteem, and suicidal thoughts and attempts. Therefore, it has a critical impact on mental health that may have worsened during a global pandemic [10]. Reduced social interaction, stay-at-home restrictions, difficulties in schoolwork, changes in daily routine, fear of illness, and boredom can have dramatic psychological effects on adolescents [11].

In general, depression, anxiety, and stress have spread among adolescents worldwide and are often diagnosed simultaneously because of their high rates of comorbidity; they are among the most common affective disorders. These mental disorders show in moderate to severe levels and are the focus of attention for those researching the psychological impacts of COVID-19 and its variants in the adolescent population. The recent alarming evidence that depression, anxiety, and stress are increasing has prompted worldwide calls for urgent measures to undertake further studies of mental disorders in adolescents during the COVID-19 pandemic [12].

The Depression, Anxiety, and Stress Scale (DASS-21) is a set of three self-report scales designed to measure the emotional states of depression, anxiety, and stress. It includes the essential symptoms of each condition and excludes those that may be present in both disorders (e.g., changes in appetite). Originally, it consisted of 42 items. However, it currently consists of 21, with distinct subscales for depression (measuring low positive affect), anxiety (measuring psychophysiological agitation), and stress (measuring negative affect). In 2014, it was adapted for Chilean high school students, and satisfactory reliability results were obtained for the depression (*α* = 0.85), anxiety (*α* = 0.72), and stress (*α* = 0.79) scales [13].

Dapieve-Patias et al. adapted and validated the DASS-21 for Brazilian adolescents (aged 12–18 years). The confirmatory factor analysis resulted in the original model being adjusted to three factors to yield optimum results. The subscales demonstrated adequate levels of internal consistency, ranging from 0.83 to 0.90 (anxiety 0.83, stress 0.86, and depression 0.90) [14].

However, information on the early detection of each psychopathological disorder among the Mexican population remains scarce. Given that the prevalence of stress, depression, and anxiety during the pandemic has significantly increased, it is crucial that countries such as Mexico—one of the countries hardest hit by COVID-19—assess stress, depression, and anxiety levels in different age groups. Such research will allow more precise diagnosis and thereby promote more effective psychological interventions.

## 2. Materials and Methods

### 2.1. Aims

The aims were to evaluate the prevalence of depression, anxiety, and stress in junior high school students in the metropolitan area of Guadalajara during the COVID-19 pandemic and to determine whether any differences in prevalence were related to sex, age group, school shift (i.e., morning or evening), school year, presence of first-degree relatives with COVID-19, and whether one or both parents worked. It is hypothesized that mental disorders will become more prevalent as a result of social changes resulting from the COVID-19 pandemic.

### 2.2. Design

A cross-sectional survey was used to evaluate the prevalence of depression, anxiety, and stress with the DASS-21 as an anonymous online survey. In addition, we asked about the participants’ sex, age group, school shift, school year, family type, whether any first-degree relatives had COVID-19, and whether either or both of their parents worked. We defined a nuclear family as a couple with children, a single-parent family as one with only a mother or father living with their children, extended family as a nuclear or single-parent family living with one or more direct bloodline family members (i.e., grandparents, uncles, cousins), and unipersonal family as those who lived alone.

### 2.3. Instrument

The Spanish version of the DASS-21 scale was used [15]. This instrument has three subscales, each comprised of seven items designed to evaluate the severity of depression, anxiety, and stress symptoms. Each item has a Likert response scale with four options. The depression subscale (items 3, 5, 10, 13, 16, 17, and 21) assesses symptoms such as sadness, anhedonia, or worthlessness. The anxiety subscale (items 2, 4, 7, 9, 15, 19, and 20) assesses symptoms such as agitation, panic attacks, fear, and physical arousal. Finally, the stress subscale (items 1, 6, 8, 11, 12, 14, and 18) assesses symptoms such as irritability, reaction to daily life or stressful events, and tension. On a scale ranging from zero to three, respondents indicate the extent to which they experienced the symptoms during the previous week. The cumulative scores for each sub-scale of depression, anxiety, and stress are calculated by summing the scores and then multiplying by two. The cut-off scores can be found in Table 1 [16]. 

### 2.4. Participants

A total of 1500 students were invited to participate from May to July 2021. A non-probabilistic purposive sampling method was used the recruit individuals studying in junior high schools in the metropolitan area of Guadalajara. The recruitment team went to six different high schools. Students were invited to answer the survey, outlining the study’s purpose and what their involvement would entail. Students were given a link to the online survey once they had consented to it. A time limit of 15 min per classroom was set, and instructions on how to complete the survey were provided. The information was gathered in a database after the participants were acknowledged by the research team. Our inclusion criteria were students from secondary schools in the metropolitan area of Guadalajara, of all school grades, who wished to participate in the study. The exclusion criteria were individuals who were not enrolled in an academic program. Incomplete surveys were excluded from the analysis. The participants answered the surveys anonymously.

### 2.5. Sample Size

The sample size was calculated with Epi Info StatCalc software (CDC, Atlanta, GA, USA), based on the total population of students at the public junior high school level in Guadalajara (73,226 students). We calculated the minimum necessary number of surveys to be 661 with an error of 5% and a confidence level of 99.99%. 

### 2.6. Data Analysis

The statistical analysis was performed using the Statistical Package for the Social Sciences (ver. 25, IBM Corp., Armonk, NY, USA) for Windows. The cumulative scores for each sub-scale were calculated by summing the scores and then multiplying by two for each participant. The descriptive analysis included proportions, means, and standard deviations. The inferential analysis was performed using the chi-squared test, Fisher’s exact test, or analysis of variance (ANOVA) test as appropriate for the categorical variables. Student’s *t* test was used to analyze continuous variables. The study variables were sex, age, grade, school shift, and history of a family member who had suffered from COVID-19. Cronbach’s alpha test was used to measure internal consistency. The probability level *p* < 0.05 was considered statistically significant.

## 3. Results

**Figure 1:**
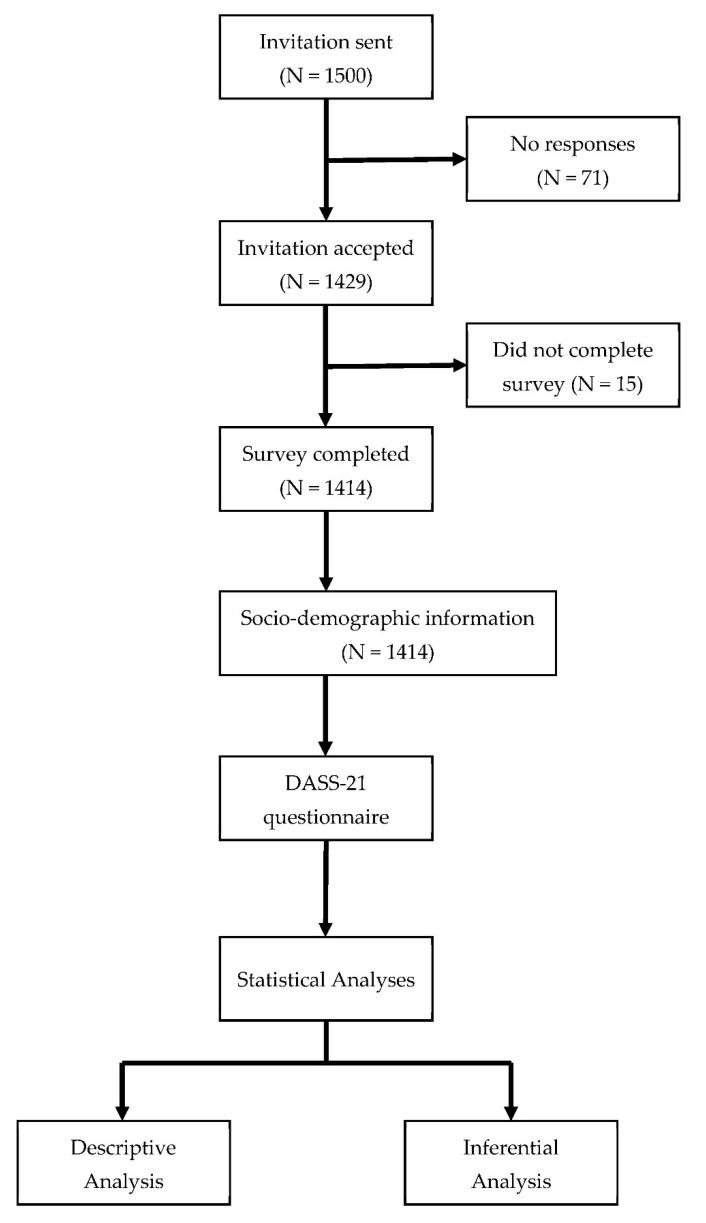
Survey flowchart.

A total of 1429 surveys were obtained, and 15 were excluded because they were incomplete. A survey flowchart is presented in Figure 1. Thus, the final sample included 1414 participants, of which 635 (44.9%) were male and 779 (55.1%) were female. More demographic characteristics are presented in Table 2.

All variables were assessed with Levene´s test for equality of variances, assuring that all variables had a parametric distribution. The Cronbach’s alpha test suggested a good internal validity for each of the subscales: depression (*α* = 0.92), anxiety (*α* = 0.89), and stress (*α* = 0.88). The DASS-21 global scores were 6.15 ± 5.6 for depression (D), 5.8 ± 5.2 for anxiety (A), and 8.08 ± 5.3 for stress (S). There were no significant differences in scores between those in the 12–13-year and 14–16-year age groups (D, *p* = 0.21; A, *p* = 0.95; and S, *p* = 0.29). Moreover, there were significant differences in the D, A, and S scores (*p* < 0.001 for each variable) between the sexes. Students from public schools had higher D, A, and S scores than students from private schools, but this difference was only significant for D (*p* = 0.004). Students who attended the morning school shift presented lower D, A, and S mean scores, but only D (*p* < 0.001) and A (*p* < 0.001) scores were statistically significant.

We compared the D, A, and S scores of the students who had one working parent with those who had two working parents and found no significant differences between them (*p* > 0.05). However, we found a statistically significant difference in A scores between students who had a relative who was infected with COVID-19 and those who did not (*p* < 0.004). Finally, there were no significant differences in D, A, or S scores between students who had an infected family member who had died and those who had not (*p* > 0.05). The remaining scores can be seen in Table 3.

A one-way ANOVA was performed to compare the differences between school grades with D, A, and S; however, no statistically significant difference was found. Finally, a one-way ANOVA was used to compare D, A, and S scores between the existing family types, and this showed a significant difference for the three items (D, *p* < 0.001; A, *p* < 0.001; S, *p* = 0.026). A post hoc multivariate analysis using the Bonferroni significance test found significantly different scores for depression between students in nuclear families and students in single-parent families (*p* = 0.037) and between students in nuclear families and students in extended families (*p* = 0.004). Students in nuclear families also showed statistically different scores for anxiety from students in single-parent families. There were no statistically significant differences in stress scores between family types in the post hoc tests (*p* = 0.001). Students in unipersonal families presented higher scores for all three variables, but these differences were not statistically significant.

## 4. Discussion

The overall DASS-21 scores were 6.15 ± 5.6 for depression, 5.8 ± 5.2 for anxiety, and 8.08 ± 5.3 for stress in our sample. Female students scored higher in all three conditions (*p* < 0.001). Students from public schools had higher depression scores than students from private schools (*p* = 0.004). Those who attended the morning school shift presented significantly lower depression (*p* < 0.001) and anxiety (*p* < 0.001) scores. Students who had a relative who was infected with COVID-19 presented higher anxiety scores than those who did not (*p* < 0.004).

The health crisis caused by COVID-19 has already had a global impact and will leave a deep and permanent mark on history. During this pandemic, physical and psychological exhaustion has been demonstrated worldwide, affecting the population regardless of their age group or social category. Although some groups are more directly exposed to the catastrophe of the pandemic, we cannot ignore what our young population has faced in terms of reduced social interaction, stay-at-home restrictions, difficulties with homework, changes in daily routine, fear of illness, and boredom, all of which can create psychological effects in adolescents. Although Duan et al. cite the lack of mental health care in the pediatric population, as one of the age groups least affected by this pandemic [17], Zhang et al. note that more than one-fifth of the sampled secondary school population in their study had their mental health affected during the pandemic [11].

It is also important to emphasize that mental disorders can appear early and are not always identified. Because psychological problems, including stress, depression, and anxiety, affect a substantial number of teenagers, the World Health Organization estimates that between 10% and 20% of adolescents worldwide suffer from mental disorders.

Furthermore, these disorders are frequently misdiagnosed or treated incorrectly. Neglecting adolescents’ and young people’s mental health can lead to suicide in the worst of scenarios. Depression is one of the leading causes of illness and disability among adolescents worldwide, with suicide being the third leading cause of death for young people aged 15–19 years and the fifteenth leading cause among those aged 10–14 years. Anxiety is the ninth most common cause of death among teenagers aged 15–19 years and the sixth in those aged 10–14 years [18].

It has been reported that adolescents with anxiety disorders find it difficult to adjust to COVID-19-related changes. For instance, those who fear COVID-19 may experience increased preoccupation with contamination and engage in excessive handwashing. Even after undergoing the quarantine period, there is still cause for concern. During the pandemic, some children with separation anxiety may become even more dependent on their caretakers, making it difficult for them to return to school [19]. In addition, infants and teens who have endured terrible times during humanitarian crises may develop sleep difficulties, anxiety, melancholy, self-harm, and difficulty concentrating.

In addition, sleep, hunger, self-care problems, isolation, apathy, and ignoring health advice are more common in adolescence [20]. Furthermore, psychological effects in infants and adolescents during pandemics have been observed. These include refusal to return to school activities, hyperactivity, irritability, loss of attention and focus, personality and behavior changes, apathy, spontaneous sobbing, and memory difficulties in adolescents aged 12–18 years [21].

In a review of mental disorders in adolescents during the COVID-19 pandemic, similar findings were observed, including an increase in stress, anxiety, sleep disorders, and depression incidence. Similar to our findings, female sexuality was identified as a risk factor. Additionally, prior mental health problems, fear of COVID-19, and listening to news concerning COVID-19 were highlighted as risk factors. [22]

The use of instruments such as the DASS-21 has been shown to help in the early diagnosis of stress, depression, and anxiety in adolescents, as noted by Andrade et al. Their study, which included factor analysis, found high internal validity for the DASS-21 when applied to adolescents [23]. A Vietnamese study also found the application of the DASS-21 in adolescents to have excellent validity, although the authors suggested re-evaluating some items to understand this age group better [24].

One study from China that surveyed 359 children and 3254 adolescents found that having a family member or friend sick with COVID-19 was associated with elevated anxiety levels, which is consistent with our findings [17]. It is clear that the COVID-19 outbreak has had a significant psychosocial impact on children and adolescents, and the current levels of anxiety and depression found highlight the need to address the emotional distress that children and adolescents experienced during the pandemic [25].

The strengths of this study include its relatively large sample size among an understudied population during a public health crisis. We presume that the strategy for administering the questionnaire, which included a face-to-face invitation and a specific time to answer the survey, increased the response rate (95.2%). Some limitations of this study include our instrument, the DASS-21, which is not specifically designed for this age group and therefore has not been validated in the pediatric population. Likewise, although our sample size exceeded the minimum required, caution is necessary in generalizing our findings to the broader population of adolescents. Our results should be interpreted under the assumption that our sample may be influenced by a non-responder bias based on students who did not agree to participate in our study.

## 5. Conclusions

The use of the DASS-2 questionnaire in different populations and circumstances provides us with information on the usefulness of this tool. It provides a precedent for evaluating student performance under diverse social circumstances. Comparing them with other studies that have incorporated similar approaches, it places them in an international context to evaluate health care policies regarding mental health. 

During the COVID-19 pandemic, physical and psychological exhaustion has been demonstrated worldwide, affecting the population regardless of their age group or social category. The pandemic and its restrictions have had a direct impact on young people’s mental health. Screening questionnaires for mental disorders can be a very useful tool in light of the changes brought by the COVID-19 pandemic. The identification of vulnerable groups is necessary to develop public health measures that benefit this population.

The data presented here and in the international literature cannot be ignored, and further research is required to develop strategies that can assist young people in coping with the changes brought about by this global crisis.

## Figures and Tables

**Table 1:** Depression, anxiety, and stress cut-off scores.

	Normal	Mild	Moderate	Severe	Extremely Severe
Depression	<9	10–13	14–20	21–27	>28
Anxiety	<7	8–9	10–14	15–19	>20
Stress	<14	15–18	19–25	26–33	>34

**Table 2:** Demographic characteristics of the sample.

Age (years), (Mean ± SD)	13.39 ± 0.99
Sex, *n* (%)	
Female	779 (55.1%)
Male	635 (44.9%)
Age group (years)	*n* (%)
12–13	788 (55.7%)
14–16	626 (44.3%)
Type of school	*n* (%)
Public	1076 (76.1%)
Private	338 (23.9%)
School shift	*n* (%)
Morning shift	1188 (84%)
Evening shift	226 (16%)
School grade	*n* (%)
1st grade	552 (39%)
2nd grade	450 (31.8%)
3rd grade	412 (29.1%)
Type of family	*n* (%)
Nuclear family	763 (54%)
Extended family	353 (25%)
Single-parent family	235 (16.6%)
Unipersonal family	63 (4.5%)

**Table 3:** DASS-21 scores by age group, sex, type of school, school shift, parental work, and family member infected with COVID-19.

Age Group	Depression Mean Scores	*p*-Value	Anxiety Mean Scores	*p*-Value	Stress Mean Scores	*p*-Value
12–13	6.27 ± 5.6	0.21 *	5.9 ± 5.2	0.95 *	8.09 ± 5.3	0.29 *
14–16	6.01 ± 5.5		5.8 ± 5.1		8.07 ± 5.4	
Sex						
Female	7.37 ± 5.9	0.001 *	7.07 ± 5.5	0.001 *	9.41 ± 5.4	0.001 *
Male	4.66 ± 4.7		4.38 ± 4.3		6.45 ± 4.7	
Type of school						
Public	6.39 ± 5.6		6.02 ± 5.1		8.11 ± 5.3	
Private	5.41 ± 5.3	0.004 *	5.36 ± 5.3	0.95 *	7.99 ± 5.3	0.91 *
School grade						
1st grade	5.76 ± 5.4	0.073 ^†^	5.54 ± 4.9	0.167 ^†^	7.82 ± 5.1	0.207 ^†^
2nd grade	6.25 ± 5.7		6.15 ± 5.4		8.44 ± 5.56	
3rd grade	6.58 ± 6.61		5.86 ± 5.21		8.08 ± 5.3	
School shift						
Morning shift	5.88 ± 5.4	0.001 *	5.65 ± 5.1	0.001 *	7.90 ± 5.3	0.37 *
Evening shift	7.59 ± 6.0		6.98 ± 5.6		9.03 ± 5.5	
Type of family						
Nuclear family	5.59 ± 5.5	0.001 ^†^	5.34 ± 5.0	0.001 ^†^	7.76 ± 5.2	0.026 ^†^
Extended family	6.58 ± 5.7		6.19 ± 5.2		8.13 ± 5.3	
Single-parent family	7.0 ± 5.69		6.91 ± 5.5		8.72 ± 5.4	
Unipersonal family	7.44 ± 5.6		6.41 ± 5.3		9.28 ± 5.7	
Parental work						
Only one parent works	5.98 ± 5.6	0.70 *	5.68 ± 5.3	0.21 *	7.96 ± 5.5	0.12 *
Both parents work	6.24 ± 5.5		5.96 ± 5.1		8.15 ± 5.2	
Infected family member						
Yes	6.39 ± 5.7	0.13 *	6.19 ± 5.3	0.004 *	8.52 ± 5.4	0.10 *
No	5.81 ± 5.4		5.39 ± 5		7.43 ± 5.1	

Notes: *: *p* value was obtained using the Students’ *t*-test. ^†^: *p* value was obtained using the ANOVA test.

## Data Availability

The datasets generated during and analyzed during the current study are available from the corresponding author upon reasonable request.
